# Prevalence of Molar–Incisor Hypomineralization and Caries in Eight-Year-Old Children in Croatia

**DOI:** 10.3390/ijerph17176358

**Published:** 2020-09-01

**Authors:** Davor Jurlina, Zvonimir Uzarevic, Zrinka Ivanisevic, Nikola Matijevic, Marko Matijevic

**Affiliations:** 1 Faculty of Medicine Osijek, University of Osijek, 31000 Osijek, Croatia; 2 Faculty of Education, University of Osijek, 31000 Osijek, Croatia; zuzarevic@foozos.hr; 3 Faculty of Dental Medicine and Health, University of Osijek, 31000 Osijek, Croatia; zrinka.ivan@gmail.com (Z.I.); marko.matijevic@fdmz.hr (M.M.); 4 School of Dental Medicine, University of Zagreb, 10000 Zagreb, Croatia; nm.matijevic@gmail.com

**Keywords:** prevalence, molar–incisor hypomineralization, Croatia, caries

## Abstract

The aim of this study was to detect molar–incisor hypomineralization (MIH) and caries prevalence in eight-year-old children with early mixed dentition in Eastern Croatia. There is a lack of data on MIH in Croatia. There were 729 children examined in total: 356 (48.83%) were female and 373 (51.16%) were male. The presence of MIH was found in 95 children, the prevalence of MIH was 13%, and the remaining 634 (87%) did not have any changes associated with MIH. The prevalence of caries overall in the population of examined children was 11.48%. In the group of children with MIH, the prevalence of caries was 24.14%, while in the group of children with no MIH, the prevalence of caries was 11.18%. Teeth had a Decayed, Missing, and Filled Teeth (DMFT) index of 1.2, the value of the SiC index was 1.4, and the decayed, missing, and filled teeth (dmft) index for deciduous teeth was 5.8. Children with MIH had a caries index of DMFT 2.1, and the value of the SiC index was 2.6. The index of caries for non-MIH children was DMFT 1.1 and the SiC index was 1.2. MIH prevalence in Croatia is more common in girls than in boys. MIH has a significant impact on children’s development and is a major factor in the occurrence of caries.

## 1. Introduction

The term molar–incisor hypomineralization (MIH) was cited and defined for the first time by Weerheijm and associates in 2001 [1,2]. The basic characteristics of MIH are the presence of white, yellow, and even brown pigmentation on the surface of the first permanent molars and on the incisors of the upper and lower jaw [3,4,5]. Pigmentation is accompanied by damage to the enamel and enamel crystals, resulting in permanent damage or even loss of the first permanent molars and incisors that are affected by MIH [6]. Changes occur to the first permanent molars and incisors in children, and the most suitable age to study this phenomenon is eight years, when there is at least one permanent molar or incisor in the mouth. This guidance is provided by the criteria advocated by the European Academy of Paediatric Dentistry (EAPD) [3]. Teeth affected with MIH have weaker enamel, and over time, the teeth become more prone to progressive action and caries, greater sensitivity, and faster decay [7]. MIH is assumed to be a consequence of environmental factors that systematically influence, among other things, the normal function of ameloblasts during the creation of the enamel [4,5]. The presence of MIH in children complicates treatment and the choice of proper therapy, and it leads to poor anesthesia tolerance, aesthetic deterioration in the forehead area, and chewing in the area of the jaw [8]. EAPD has a specific approach to the selection of criteria for review and registration, but when MIH is investigated, there are various methods used, and the results can be interpreted differently. The best time to record MIH is eight years old, when there are permanent incisors and the first permanent molars are in the mouth. Prevalence of MIH in the literature ranges from 2.4% to 40.2%, and each study uses different criteria [9,10,11,12,13,14,15,16,17,18].

The Republic of Croatia (RH) is a European country, a member of the European Union, located geographically in the southern part of Central Europe and the northern part of the Mediterranean. Studies on the frequency of MIH as well as its association with the occurrence of caries in the territory of the Republic of Croatia have not been carried out yet. Without research and outcomes, there is no basis to educate parents, inform Doctor of Dental Medicine programs, or to advance the recognition and prevention of MIH in the Croatian population. The aim of this study is to detect MIH and caries prevalence in eight-year-old children with early mixed dentition in Eastern Croatia.

## 2. Materials and Methods

### 2.1. Description of the Research Domain

The Republic of Croatia is a member of the European Union, and according to the latest measurements in 2011, it has about 4,284,889 inhabitants. The current research was conducted in randomly selected primary schools in the eastern part of Croatia. The exploration area included urban and rural areas, which differ in terms of health care availability, education, nutrition, and awareness of the importance of oral health to both parents and children.

### 2.2. Procedure

Prior to joining the Clinical Examination of Children, approval was obtained from the Ethics Committee of the Osijek Medical School at the Josip Juraj Strossmayer University in Osijek, and consent was obtained from the principals of the 11 elementary schools included in this study. 

To conduct this research, licensing from the Ethical Committee of the Faculty of Medicine at the University of Osijek was obtained (Class: 602-04/15-08/08; No. 2158-61-07-15-127), in accordance with the Helsinki Declaration on Medical Research Involving Human Subjects. All procedures were explained clearly to the parents/guardians and participants before their inclusion in the study. Informed consent to oral examination was obtained from all the parents/guardians of the children. The methods employed were applied in accordance with approved guidelines.

Upon approval from the Ethics Committee, approvals and questionnaires were sent to the parents or guardians of the children. Upon receipt of the signed and completed approvals and questionnaires, primary school principals contacted the examiner (D.J.) to organize clinical examinations for the children.

Clinical examinations were conducted over a period of two years and included only children that were eight years old, who had a completed a questionnaire, and had signed consent from their parent or guardian; otherwise, children were excluded from the survey. Other exclusion criteria were as follows: ill on the day of examination, absent from school, or afraid of a dentist and a noninvasive examination. The examiner explained in detail the inspection protocol as well as the purpose of the research to parents so they could be fully informed.

### 2.3. Training of the Examiner

Prior to conducting clinical examinations, the examiner (D.J.) was trained in order to adequately identify the changes required on the teeth. Training was performed via clinical examination of five children in MIH therapy aged 9–12 years with the occurrence of MIH. A large number of photographs showing different stages and localization of MIH were also obtained from other examiners and from professional literature under the control and supervision of a specialist in pedodontics from the Department of Dental Medicine Osijek, according to the EAPD. A second training was conducted after a few weeks as a control with the addition of photographs that did not show MIH, about which the examiner did not know. Training was deemed successful when the minimal percentage of error (the kappa value) was 0.95. A training supervisor was present during the first examinations to ensure standardized diagnosis and recording.

### 2.4. Inclusion and Exclusion Criteria

Inclusion Criteria for Access to Review included the following:

- All children aged eight years, from the eastern part of the Republic of Croatia, with signed consent of the parent or guardian.

Exclusion Criteria for Access to Review included the following:

- Children whose parents did not give written consent;
- Children who were ill on the day of the examination or who, for some other reason, were absent from school;
- Children who were afraid of the dentist, with signed consent of their parents;
- Children in orthodontic therapy with fixed orthodontic aids.

### 2.5. Review Layout

Prior to examination, all children were instructed to brush their teeth. Those who did not brush were instructed to wash their mouth several times with a large amount of water. 

The examination was carried out in well-lit rooms (both natural and artificial light). For better illumination, as an additional source of light, the examiner wore an LED light bulb on the forehead to aid in detecting MIH.

The children were examined in alphabetical order, and each child was tabulated according to their MIH stage, oral status, and presence of caries in permanent and deciduous dentition.

A single sterile dental mirror and a dental probe were used in the examination. Recognition of MIH was recorded and ranked according to the criteria from the EAPD and adapted from Ghanim and associates in 2011 [16,19,20]. Results were illustrated and stored digitally according to the signed consent.

The EAPD criteria for determining MIH are ranked as follows: 0—enamel without defect; 1—white/creamy limited areas of opacity without posterity loss of the enamel; 1a—white/creamy limited areas of opacity with posterity loss of enamel; 2—yellow/brown limited areas of opacity without posterity loss of enamel; 2a—brown/brown limited areas of opacity with posterity effect loss of enamel; 3—atypical restorations; 4—tooth loss due to MIH; 5—partially projected teeth (less than one-third of the dental crown) with MIH presence; 6—absent/partially released teeth without MIH presence; 7—diffuse opacity (not MIH); 8—hypoplasia (not MIH); 9—combined changes (diffuse opacity/hypoplasia with MIH); 10—limited opaque areas only on the incisors (Table 1) [9,21,22].

All observed changes less than 2 mm were not recorded as possible MIH. There were no cases of extracted teeth, so missing teeth could not be recorded as a result of MIH presence.

Data were analyzed by excluding MIH characteristics from Table 1, which implies the exclusion of Stages 1, 1a, 2, 2a, 3, and 4. However, no Stage 4 or extracted teeth were found in the surveyed population because the group of eight-year-olds had no possibility of losing permanent teeth caused by MIH; thus, this stage was not included in the statistical analysis.

We analyzed data according to incisor hypomineralization (IH), molar hypomineralization (MH), and standard MIH.

By clinical examination, IH can show the presence of MIH, but this does not have to be due to the morphological similarities of the hypomineralization of central incisors in other enamel abnormalities; thus, for precise MIH diagnosis, it is necessary to have at least one permanent molar with MIH characteristics.

MH is a more credible defect that gives us greater certainty of MIH diagnosis due to the stronger features of typical hypomineralization and decay of the enamel in accordance with MIH stages. The MIH value was determined for children with clearly developed molars and incisors, thus confirming MIH presence, but the total number of MIHs also included children with IH; therefore, the sum of MIH and IH was used to determine MIH prevalence.

To estimate the prevalence of carries, the usual DMFT (Decayed, Missing, and Filled Teeth) indicator was used, which is an index showing the presence of dental caries in permanent dentition of the subject and is expressed numerically as the sum of the caries (D) and the extracted (M) teeth treated by filling (F); a similar index is dmft (decayed, missing, and filled teeth), which can assess the condition of deciduous dentition. The Significant Caries Index focuses on people with the highest DMFT index in the population, representing on average about 1/3 of the population [23,24,25,26].

### 2.6. Respondents

A total of 729 eight-year-olds (356 female and 373 male, average 8.21 ± 0.12 years) met the inclusion criteria. Respondents were from the urban and rural areas of Eastern Croatia. The identities of the respondents were protected (encrypted). Data were processed after clinical examination, and subjects were classified in total, by gender, in the group with MIH, and in the group without the presence of MIH (non-MIH).

The sample size was obtained by calculating the proportional sample size (relative error). The planned population size was 1383, with a desired reliability of 0.95 and error boundaries of 0.05, and the expected ratio was 0.50. A sample size of 729 was obtained, and this is within the upper limit of the sample size or number of MIH research subjects recommended by the EAPD according to Weerheijm in the latest guidelines [27].

### 2.7. Statistical Methods

The data collected during the research were stored in a database in Microsoft Office Excel 2003 (Microsoft, Redmond, WA, USA) and processed by a personal computer by statisticians. Basic statistical descriptive methods were applied. For MIH differences between sexes, a Chi-Squared test at the 0.05 significance level was applied. Confusion matrices for different comparisons were built including information on true negative, false negative, false positive, and true positive as well as measures of specificity, sensitivity, positive prediction value, negative prediction value, and the odds ratio. Distribution and prevalence of specific conditions were also provided. Finally, in analyzing the posteruptive damage of enamel (PEB), the chance ratio and its 95% interval of reliability were estimated.

## 3. Results

The number of examined eight-year-olds included in this study was 729, out of which 356 (48.8%) were female and 373 (51.2%) male.

In the total number of children surveyed, the DMFT index was found to be 1.2 and the SiC index 1.4, while the dmft index was 5.8.

The 729 children were grouped into MIH (*n* = 95, 13%) and non-MIH (*n* = 634, 87%).

The total prevalence of MIH was 13%.

In the group of 95 children with MIH, 56 (15.7%) were female and 39 (10.5%) male.

The DMFT index value for children with MIH was 2.1, and the SiC index was 2.6.

In the group of 634 children without MIH, 300 (84.3%) were female and 334 (89.5%) male.

The DMFT index value for children without MIH was 1.1, and the SiC index was 1.2.

### 3.1. Possible Influences of Dmft or DMFT Status on MIH Occurrence

Dmft analysis revealed a very low correlation between dental caries and appearance of MIH (odds ratio: 1.59), which is shown in detail in Table 2. Influence of permanent tooth caries (i.e., DMFT) on MIH appearance was significantly impacted by DMFT (odds ratio: 5.06), as described in Table 3.

Measurements were as follows: specificity SP = TN/(TN + FP) = 11.2%, sensitivity SE = TP/(TP + FN) = 92.6%, positive predictive value PPV = TP/(TP + FP) = 13.5%, negative predictive value NPV = TN/(TN + FN) = 91.0%, and odds ratio = (TP/FP)/(FN/TN) = 1.59.

Measurements were as follows: specificity SP = TN/(TN + FP) = 50.6%, sensitivity SE = TP/(TP + FN) = 83.2%, positive predictive value PPV = TP/(TP + FP) = 20.2%, negative predictive value NPV = TN/(TN + FN) = 95.3%, and odds ratio = (TP/FP)/(FN/TN)) = 5.06.

### 3.2. Distribution and Prevalence of Hypomineralization Lesions in Permanent Teeth in Eight-Year-Olds

By analyzing the selected criteria in Table 4, it can be seen that MIH in one or more incisors was present only in girls, and the presence of MIH in one or more molars was also higher in girls than in boys. At least one molar and at least one incisor with MIH were present in equal numbers in girls and boys. The total number of girls with MIH and IH was significantly higher than that of boys (Chi-Squared = 4.4714, *p* = 0.0345). In total, there was a greater presence of MIH and IH in girls than boys.

MIH classified by EAPD stage was high in both stages without cavitation (in the amount of 22.1%) and with cavitation (in the amount of 61.1%), while the percentage of atypical restorations was 15.8%.

The appearance of white creamy limited opacities without cavitation was 6.3%, and white creamy with cavitation was 23.2%. Yellow brown with limited opacity without cavitation was 15.8%, and with cavitation was 38.9%, which indicated a higher degree of yellowish brown, limited opacity without and with cavitation on the affected tooth.

Statistical data were processed for teeth and MIH stages as follows: Incisors: 1.1, 1.2, 2.1, 2.2, 3.1, 3.2, 4.1, 4.2; Molars: 1.6, 2.6, 3.6, 4.6; MIH stages: 1, 1a, 2, 2a, 3.

### 3.3. Number of Affected Molars and Incisors in Hypomineralization Lesions in Eight-Year-Olds Classified into Subgroups

Children were classified according to the number of affected molars and incisors in all 12 teeth (i.e., upper and lower permanent incisors, upper and lower permanent first molars), and groups with affected incisors emerged in all and the first 4 permanent molars (Table 5).

In the second column, children with all 12 index teeth (1.1, 1.2, 2.1, 2.2, 3.1, 3.2, 4.1, 4.2 and 1.6, 2.6, 3.6, 4.6) were considered. This subset contained a total of 340 children. Under this subset, children with 1, 2, 3, and 4 molars (1.6, 2.6, 3.6, and 4.6) with MIH condition 1, 1a, 2, 2a, and 3 were additionally isolated. Similar to the third column, children were considered who had sprung all four molars (1.6, 2.6, 3.6, and 4.6), and this subset contained 681 children. Our results show that a greater number of affected molars were associated with a greater number of affected incisors in both subgroups of children.

### 3.4. PEB and Opacities Linked to the Number of Affected Permanent Teeth Classified by Sex

Destruction of the dental crown due to MIH after or in the period of tooth eruption was compared between boys and girls and is interpreted in Table 6. We found similar values in the subgroups of children with MIH + IH and MIH + MH, as previously mentioned. Children in the subgroup MIH + IH without the appearance of PEB were more numerous than those in the MIH + MH subgroup, whereas in the case of the occurrence of PEB, both groups had the same number. Girls in the MIH + IH group had higher PEB numbers than boys in the same group and had a higher absence of PEB. There was a statistically significant difference between sexes related to MIH for stages 1.1a, 2.2a, and 3 (Chi-Squared = 4.4714, *p* = 0.0345).

The presence of MIH in Eastern Croatia is not negligible, and future research is needed throughout the country to gain a more complete picture of MIH’s representation in the Republic of Croatia.

## 4. Discussion

This study on the prevalence of MIH in the Republic of Croatia was conducted with 729 eight-year-olds from randomly selected elementary schools in urban and rural environments. According to the results, MIH can be characterized as a quiet enemy of children’s teeth, which, if not recognized early in life, will disturb the general health and social–economic development of the child compared with other children not affected by MIH. The selection of respondents was in line with EAPD regulations [27] that recommend the age of eight years, which is considered to be the age when children develop their first permanent molars and permanent incisors. Subjects were classified according to sex and compared to each other, and mutual comparisons were made between individual teeth, overall dental status, and the degree of MIH involvement. The DMFT index [23,24,25,26] and the MIH criteria for MIH stages were used to record the caries, with a focus on acute, limited pigmentation with and without loss of enamel, as well as the atypical restorations preceded by MIH [9,21,22]. 

The survey found that MIH prevalence was 13%. Of those with MIH, 41.4% were male and 58.9% were female. This is comparable to all other studies published so far, where MIH prevalence ranged from 2.4% to 40.2% (e.g., Lithuania 14.9%, Sweden 3.5%, Bulgaria 3.6%, Argentina 15.9%, Brazil 40.2%, Jordan 17.6% [9], Greece 10.2% [12], Germany 14.3% [13], Spain 17.8% [14], Uruguay 6.4% [15], Iraq 21.5% [16], and Finland 17.1% [17]). Our results were very close to those in neighboring countries such as Slovenia 21.4% [10] and Bosnia and Herzegovina 12.3% [11], but the differences among all the studies published so far include the age of the examinees and the size of the sample.

The research carried out by Groselj and Jan (2013) [10] in Slovenia for children aged 6–11 years found a prevalence of 21.4%, confirming the increased presence of caries among children with MIH. Muratbegovic et al. (2007) [11] found in Bosnia and Herzegovina an MIH prevalence of 12.3% in children aged 12 years, with a moderately increased caries incidence. In Kosovo, the prevalence of MIH was found to be 12.2% for the ages of 9–10 years, as investigated by Martinovic et al. (2017) [28], but they did not compare the effect on caries occurrence. In Iran, a prevalence of 12.7% was observed in children 7–9 years old, and a greater prevalence of caries was reported [29].

This study establishes a possible association of MIH with DMFT and dmft, where children with MIH have a higher DMFT value compared with non-MIH children. We found a high number of caries represented in the MIH population with both permanent and deciduous teeth. At the age of eight years in both female and male children, a greater number of molars affected by MIH was observed, with 4.4% having MIH in more than one molar compared with 1.1% having it in only one molar. This is a large difference in the number of affected teeth compared with findings from Ghanim et al. (2011) [16], who found MIH was more frequent in one molar compared with more than one molar. When comparing the obtained results for incisors in the same position, there was a higher MIH occurrence in more engorged incisors than merely in one incisor. Guided by further comparisons with Ghanim’s research in 2011, our study found a lower prevalence of two molars affected by MIH, where Ghanim found a proportional increase in the magnitude of one to four molars in children with permanent molars and incisors.

The results obtained by the EAPD-induced peripheral neoplasms or incisors showed that the incidence of cervical lesions in girls was 52.1%, compared with 47.9% in boys, while damaged enamel was higher in girls (81.8%) than in boys (18.2%). The study carried out by Ghanim found the abovementioned values in favor of boys. We conclude this difference was due to the greater number of boys that were examined in Ghana [16], while in this research, there was a larger number of girls. There is no clear conclusion that greater occurrence of MIH is associated with a particular gender.

In the study, the data on the presence of a single MIH stage were obtained, where the most represented stage was yellow/brown restricted areas of opacity with posteruptive loss of enamel (38.9%), and the least represented stage was white/creamy limited areas of opacity without posteruptive loss of enamel (6.3%). Atypical restoration in patients with MIH was 15.8%. The data indicate poor repair of teeth affected by MIH with loss of enamel, increased caries progression, as well as faster tooth loss in the sense of pulling out the affected teeth. The most commonly affected tooth according to the criteria characterizing MIH (mentioned in Table 1) was the lower left molar, numerical mark 36, with a percentage of 9.9%. The most represented MIH criterion was the yellow-brown stage with a posterity loss of enamel on tooth 36 (54.3%). Teeth affected by MIH and remedied by atypical restorations were equal between the lower right and left molars, marks 36 (50%) and 46 (50%), while most atypical restorations were on the upper right molar 16 (59.3%). The upper central left incisor 21 was most affected by white creamy limited opacities without loss of enamel (30.8%).

It is necessary to carry out future studies and compare them with the same criteria in this study in order to maintain consistency and have credible and equally comparable data.

## 5. Conclusions

The presence of MIH in Eastern Croatia is not negligible, which shows the seriousness of the problem, and more research is required on this topic in order to improve the oral health of children. The correlation between MIH and caries suggests that caries are more represented in children with MIH and that these teeth tend to develop caries faster, which can result in tooth loss. Timely education for dental practitioners and parents, in a preventive sense, can contribute to longer-term preservation of the affected teeth. Due to this previously unexplored area, and this study being the first to give data for the Republic of Croatia, it is concluded that this research is significant and greatly contributes the promotion, encouragement, and improvement of oral health, both for children and the overall population.

## Figures and Tables

**Table 1:** Criteria for determining molar–incisor hypomineralization (MIH) according to the European Academy of Paediatric Dentistry (EAPD).

Mark	Criterion
0	Enamel without defect
1	White/creamy limited areas of opacity without posteruptive loss of enamel
1a	White/creamy limited areas of opacity with posteruptive loss of enamel
2	Yellow/brown limited areas of opacity without posteruptive loss of enamel
2a	Yellow/brown limited areas of opacity with posteruptive loss of enamel
3	Atypical restorations
4	Tooth loss due to MIH
5	Partially erupted teeth (less than one-third of the dental crown) with the MIH present
6	Unerupted/partially erupted teeth without MIH
7	Diffuse opacity (not MIH)
8	Hypoplasia (not MIH)
9	Combined changes (diffuse opacity/hypoplasia with MIH)
10	Limited opacity areas only on incisors

**Table 2:** Relationship between deciduous teeth (decayed, missing, and filled teeth; dmft) and MIH.

		MIH on 16, 26, 36, 46 and 12, 11, 21, 22, 32, 31, 41, 42		
		No	Yes	
dmft on 55, 54, 64, 65, 75, 74, 84, 85	No	TN = 71	FN = 7	78
	Yes	FP = 563	TP = 88	651
		634	95	*n* = 729

MIH: molar-incisor hypomineralization, TN: true negative, FN: false negative, FP: false positive, TP: true positive.

**Table 3:** The relationship between permanent tooth status Decayed, Missing, and Filled Teeth (DMFT) and MIH.

		MIH on 16, 26, 36, 46 and 12, 11, 21, 22, 32, 31, 41, 42		
		No	Yes	
DMFT on all 28	No	TN = 321	FN = 16	337
	Yes	FP = 313	TP = 79	392
		634	95	*n* = 729

DMFT: Decayed, Missing, and Filled Teeth, MIH: molar-incisor hypomineralization, TN: true negative, FN: false negative, FP: false positive, TP: true positive.

**Table 4:** Distribution and prevalence of hypomineralization lesions in permanent teeth.

	Total *n* (%) *n* = 729	Girls *n* (%) *n* = 356	Boys *n* (%) *n* = 373
One incisor (only)	2 (0.3)	2 (0.6)	0 (0.0)
More than one incisor (only)	5 (0.7)	5 (1.4)	0 (0.0)
One molar (only)	8 (1.1)	5 (1.4)	3 (0.8)
More than one molar (only)	32 (4.4)	20 (5.6)	12 (3.2)
Molars and incisors	48 (6.6)	24 (6.7)	24 (6.4)
Total children with MIH	88 (12.1)	49 (13.8)	39 (10.5)
Total children with MIH and incisor hypomineralization	95 (13.0)	56 (15.7)	39 (10.5)

**Table 5:** Number of affected molars and incisors in hypomineralization lesions in eight-year-olds classified into subgroups.

Number of Affected Molars (1.6, 2.6, 3.6, 4.6)	Mean Number and Standard Deviation of Affected Incisors (1.1, 1.2, 2.1, 2.2, 3.1, 3.2, 4.1, 4.2) in Children with 12 Index Teeth Erupted	Mean Number and Standard Deviation of Affected Incisors (1.1, 1.2, 2.1, 2.2, 3.1, 3.2, 4.1, 4.2) in Children with 4 Molars Erupted
1	0.5 (0.9)	0.5 (0.8)
2	0.4 (0.7)	0.7 (1.2)
3	1.7 (1.8)	1.1 (1.5)
4	2.0 (2.1)	2.1 (2.2)

**Table 6:** Posteruptive damage of enamel (PEB) and opacities linked to the number of affected permanent teeth classified by sex.

	On at Least One Incisor/Molar PEB	No Appearance of PEB	Total with and without PEB	Chance Ratio	95% Interval of Reliability
Number of children with MIH + IH on ≥ 3 teeth	59 (80.8)	9 (40.9)	68 (71.6)	6.09	2.17–17.06
Number of children with MIH + IH on 1 or 2 teeth	14 (19.2)	13 (59.1)	27 (28.4)		
Number of children with MIH + IH > 0	73 (76.8)	22 (23.2)	95		
Number of children with MIH + MH on ≥ 3 teeth	67 (91.8)	11 (73.3)	78 (88.6)	4.06	0.98–16.75
Number of children with MIH + MH on 1 or 2 teeth	6 (8.2)	4 (26.7)	10 (11.4)		
Number of children with MIH + MH > 0	73 (83.0)	15 (17.0)	88		
Number of boys with MIH + IH > 0	35 (47.9)	4 (18.2)	39 (41.1)	4.14	1.28–13.44
Number of girls with MIH + IH > 0	38 (52.1)	18 (81.8)	56 (58.9)		

IH = incisor hypomineralization; MH = molar hypomineralization.
